# Case Report: Giant sacrococcygeal teratoma in neonates—a report of three cases

**DOI:** 10.3389/fped.2025.1600920

**Published:** 2025-07-22

**Authors:** Letu Wu Ri Ni, Yimin Nong, Jinben Wang, Xiujian Liang, Baoxin Zhang, Zhenying Lin

**Affiliations:** Department of Pediatric Surgery, Baoan Women’s and Children’s Hospital, Shenzhen, China

**Keywords:** sacrococcygeal teratoma, neonates, case report, comprehensive contingency plans, interdisciplinary collaboration

## Abstract

Although most cases of neonatal sacrococcygeal teratoma are benign, some can grow into very large lesions, potentially causing high-output heart failure, massive bleeding, disseminated intravascular coagulation, and even fatal outcomes. We report three cases of giant sacrococcygeal teratomas, detailing their diagnostic and therapeutic management. We emphasize that clinicians should be prepared with comprehensive contingency plans and that interdisciplinary collaboration is essential to enhance patient survival rates and minimize potential complications.

## Introduction

Teratoma is the most common germ cell tumor in children, characterized by the abnormal development of tissues derived from at least two of the three germ layers. Teratomas can occur in both the gonads and extra-gonadal sites, with a predilection for midline locations, among which the sacrococcygeal region is the most common site in neonates. Sacrococcygeal teratoma (SCT) is a tumor that arises in the sacrococcygeal region, characterized by the presence of tissue components from multiple embryonic germ layers (1).

Several factors have been identified as predictors of a poor prognosis, including tumor volume and the proportion of its vascular and/or solid components, changes in placental size and morphology, histological features of the tumor, intrapelvic extension of the tumor, a tumor diagnosis before 20 weeks of gestation, fetal hydrops, polyhydramnios, low Apgar score at birth, low birth weight, and preterm delivery before 30 weeks of gestation (2). Most fatalities are associated with high-output cardiac failure, which leads to fetal hydrops from increased vascular flow to the solid components of the tumor, or with severe hemorrhage due to tumor rupture during delivery (3). Although most sacrococcygeal teratomas in neonates are benign, some may grow to a large size and cause serious complications, including high-output heart failure and massive hemorrhage. More than 80% of fetuses with a tumor-to-fetal weight ratio (TFR) greater than 0.12 before 24 weeks of gestation suffer from poor outcomes (4). Even after surgical resection of the tumor, recurrence, malignant transformation, and other conditions may still occur in some cases during long-term follow-up (5).

There are limited reports in the literature on giant sacrococcygeal teratomas. Therefore, we report the diagnostic and treatment experiences from three cases of giant sacrococcygeal teratomas.

## Case presentation

The mothers in cases 1, 2, and 3 were 35, 32, and 34 years old, respectively. Both parents of each child were in good health, with no history of tumors or mental illness, and there was no consanguinity. The families maintained harmonious relationships. In case 1, the mother received dexamethasone to promote fetal lung maturity and magnesium sulfate for pregnancy maintenance prior to a cesarean section. The mothers in cases 2 and 3 did not receive any special medications during pregnancy. The newborn in case 1 presented with weak spontaneous respiration and hypotonia at birth. Endotracheal intubation and resuscitation using a T-piece resuscitator were performed. His Apgar scores were 8, 9, and 9 at 1, 5, and 10 min, respectively. The newborns in cases 1 and 2 exhibited stable vital signs at birth, with no signs of respiratory distress, cyanosis, grunting, or apnea. The Apgar scores were 10 at 1, 5, and 10 min.

The treatment protocol followed in case 1 is provided here as an example of the treatment protocol for a large teratoma. After a prenatal color Doppler ultrasound revealed a large fetal teratoma, obstetricians, neonatologists, and pediatric surgeons performed assessments and monitored the pregnancy to determine the optimal delivery time. Following birth, neonatologists and pediatric surgeons promptly conducted a brief assessment and transferred the infant to the neonatal intensive care unit. Prior to surgery, neonatologists, pediatric surgeons, anesthesiologists, and radiologists held a multidisciplinary consultation and completed thorough preparations. Postoperatively, the pediatric surgeon and neonatologist jointly formulated a rehabilitation plan for the infant.

The patient was placed in the prone position during the surgery. One of the challenges associated with this positioning under anesthesia is ensuring adequate respiratory support. To address this, reinforced endotracheal tubes were utilized. Throughout the procedure, the patient's vital signs and respiratory parameters were continuously monitored to allow for the prompt detection of and adjustment for any abnormalities. Immediate interventions were undertaken if any issues arose. In addition, due to the fragility of neonatal skin, a surgical posture mat and artificial skin were used to protect all areas in contact with the operating surface. The patient's head was rotated regularly, and their skin condition was assessed periodically throughout the procedure. Due to the presence of a significant lesion in the sacrococcygeal region, nerve blocks were not feasible. Therefore, postoperative pain management was primarily achieved through local infiltration anesthesia at the incision site and patient-controlled intravenous analgesia (PCIA). Fentanyl was used as the primary analgesic agent. PCIA is programmed by the anesthesiologist at a specific rate to continuously deliver pain-relieving medication, thereby achieving effective analgesia. Postoperatively, if medical staff observe signs of discomfort in the neonatal patient and believe the discomfort is due to surgical wound pain, they may activate the patient-controlled analgesia (PCA) pump to administer an additional dose of medication.

To prevent surgical site infection, prophylactic antibiotics were administered 30 min prior to the operation. Operative time was minimized, and intraoperative normothermia was maintained. Postoperatively, the patient was placed in alternating prone and lateral positions. A rectal tube was inserted after surgery and is typically removed after the first bowel movement, usually on postoperative day 2. A urinary catheter was also placed and is generally removed between postoperative days 3–5. Perianal hygiene was maintained, with care taken to keep the area clean and dry. All surgical incisions healed without complications.

### Case 1

**Figure 1:**
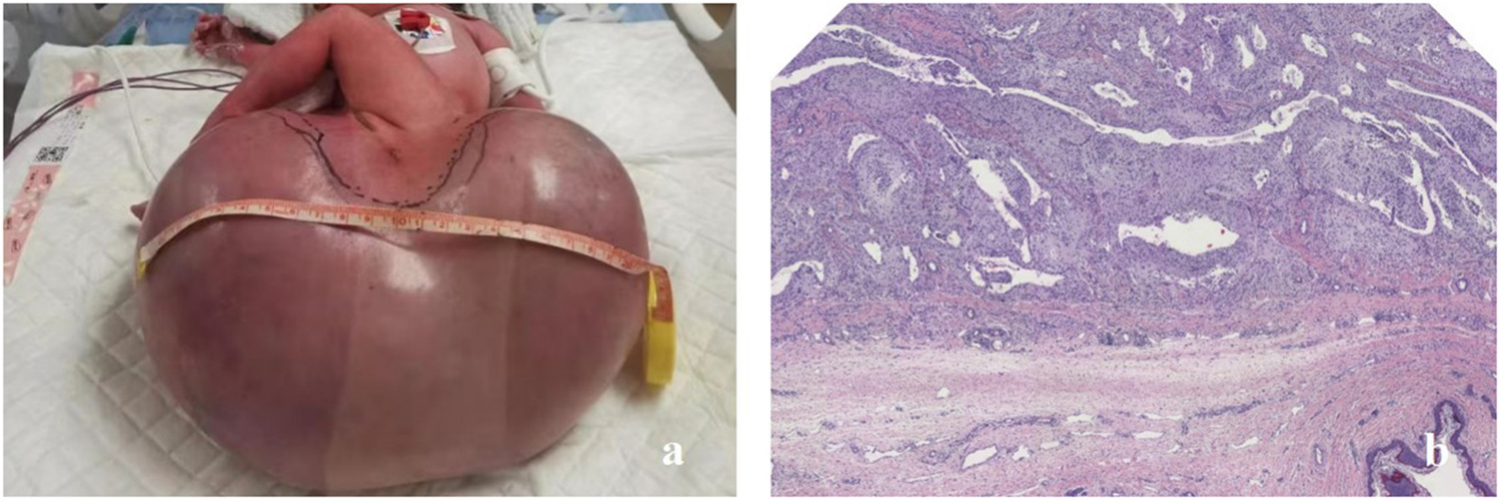
**(a)** A neonate in case 1 presenting with a giant sacrococcygeal teratoma. **(b)** The postoperative histopathological examination confirmed it to be a giant mature teratoma.

Two months before the child's birth, a three-dimensional ultrasound revealed a predominantly cystic-solid mass in the sacrococcygeal region of the fetus. There was vaginal bleeding observed prenatally. An ultrasound showed a sacrococcygeal teratoma measuring approximately 22.3 cm × 15.7 cm × 15.7 cm, with complete anterior placenta previa. After a multidisciplinary discussion, a cesarean section was performed. A large mass was observed in the sacrococcygeal region (Figure 1a). A CT scan showed a large cystic-solid mass in the pelvic floor, predominantly cystic in nature, with internal septations. The lesion's edge contained solid components, with punctate calcifications visible within the solid parts. The mass appeared to connect to the right side of the pelvis, measuring approximately 24.0 cm × 16.9 cm × 17.5 cm. After a multidisciplinary consultation, a resection of the sacrococcygeal teratoma was performed on the first day after birth.

A circumferential incision was made along the transverse axis of the mass, and the skin was incised to expose the lesion. The mass appeared cystic with a thin wall, containing fluid, gelatinous material, and firm tissue components. A resection was carried out along the subcutaneous plane to the tumor base. On the right side, the lesion extended near the right hip joint, and the joint capsule was identified. Anteriorly, the mass extended to the rectal wall. A careful resection was performed, guided by the anal canal. Posteriorly, the mass extended superiorly along the anterior aspect of the coccyx. The coccyx tip was resected. The upper pole of the mass was located approximately 3 cm from the coccyx tip, near the peritoneum. Both blunt and sharp resection techniques were used to separate the tumor from surrounding tissues, and the lesion was completely excised. The postoperative pathology results showed that it was a giant mature teratoma (Figure 1b).

**Figure 2:**
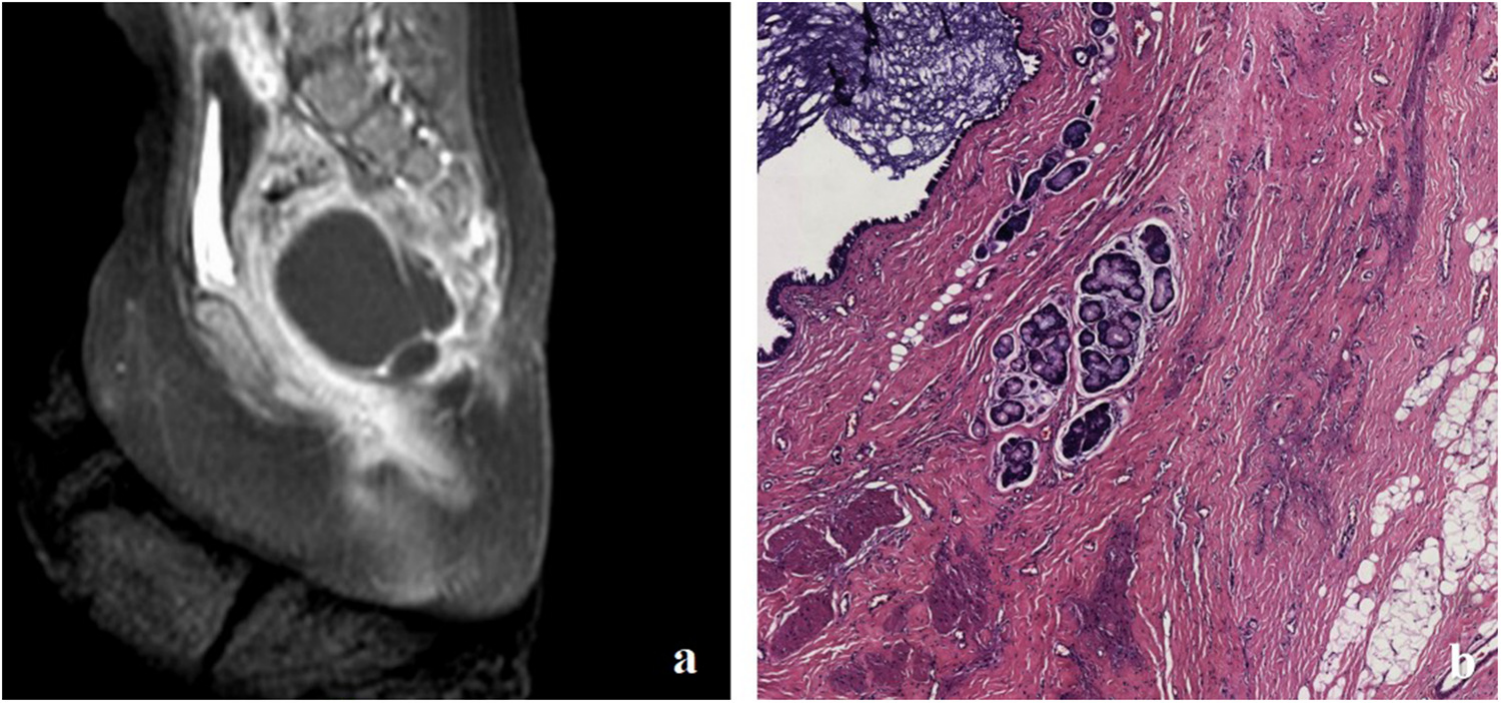
**(a)** MRI showing a cystic lesion located anterolaterally to the sacrococcygeal area in case 1. **(b)** Postoperative pathology confirmed the diagnosis of a mature cystic teratoma.

Before the operation, the patient’s alpha-fetoprotein (AFP) levels were >3,000.0 µg/L. The patient’s AFP levels remained >3,000.0 µg/L 17 days after surgery, and 1 year later, their AFP levels decreased to 3.5 µg/L. However, a rectal examination revealed a palpable, cystic mass measuring approximately 3.0 cm × 4.0 cm in the anterior sacrococcygeal area, with a smooth surface and no tenderness. Both plain and enhanced magnetic resonance imaging (MRI) scans showed a cystic lesion anterior to and to the left of the sacrococcygeal area (Figure 2a).

Follow-up MRI scans, both plain and enhanced, conducted 1.5 years after surgery, showed a slight shrinkage of the tumor; a second surgical intervention was performed to complete the excision of the cystic mass, which measured approximately 3 cm × 3 cm × 3 cm and had a thin wall. Upon opening the cystic mass, clear yellow-green fluid mixed with a milky white substance was observed. No solid tissue was found. Postoperative pathology confirmed a cystic mature teratoma without immature components (Figure 2b). Pelvic MRI scans 2 years after the second surgery showed that the cystic lesion in the left anterior sacrococcygeal area essentially disappeared.

### Case 2

**Figure 3:**
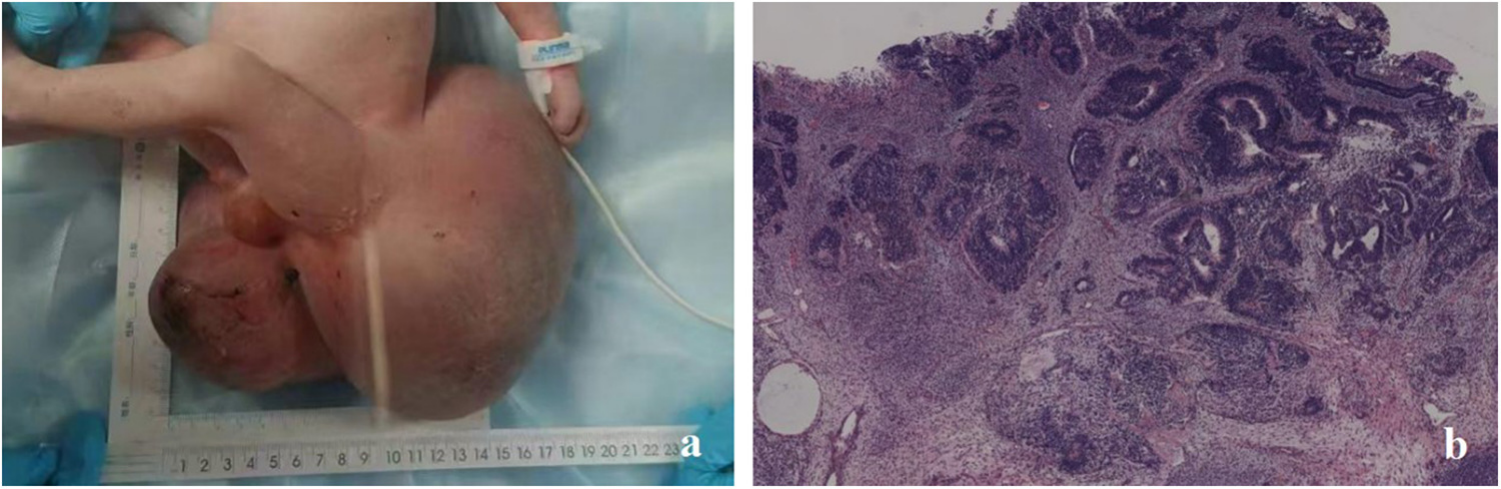
In case 2, **(a)** a neonate presented with a giant sacrococcygeal teratoma. **(b)** Postoperative pathology confirmed it to be an immature teratoma.

The infant was born with a gestational age of 32 weeks and 6 days, and a birth weight of 3,680 g. A mass measuring approximately 20 cm × 16 cm × 11 cm was observed in the sacrococcygeal region on imaging (Figure 3a). The patient’s AFP levels were >192,000.0 µg/L. MRI of the sacrococcygeal region showed a large cystic-solid mass in the buttocks and perineum, extending into the pelvic cavity with a rich blood supply. A resection of the sacrococcygeal teratoma and partial coccygectomy with drainage was performed on the second day after birth.

The patient’s preoperative hemoglobin concentration (HGB) was 125 g/L, and 170 mL of washed red blood cell suspension was transfused. During surgery, significant bleeding from the wound occurred, with an estimated intraoperative blood loss of approximately 300 mL. In total, 200 mL of red blood cell suspension and 100 mL of plasma were transfused intraoperatively. On the first postoperative day, a small amount of persistent wound bleeding was observed. Laboratory tests showed a prothrombin time (PT) of 20.3 s, an international normalized ratio (PT-INR) of 1.78, an activated partial thromboplastin time (APTT) of 64.9 s, a fibrinogen (FIB) level of 1.03 g/L, and a HGB of 119 g/L. Subsequently, an additional 85 mL of washed red blood cells, 43 mL of platelets, and 42 mL of plasma were transfused.

The postoperative pathological result was immature teratoma, grade 3 (the immature teratoma grading was based on the amount of neuroepithelial tissue) (Figure 3b). The patient’s postoperative AFP level gradually decreased. No abnormalities were observed in the sacrococcygeal ultrasound performed at 4 months postoperatively.

### Case 3

**Figure 4:**
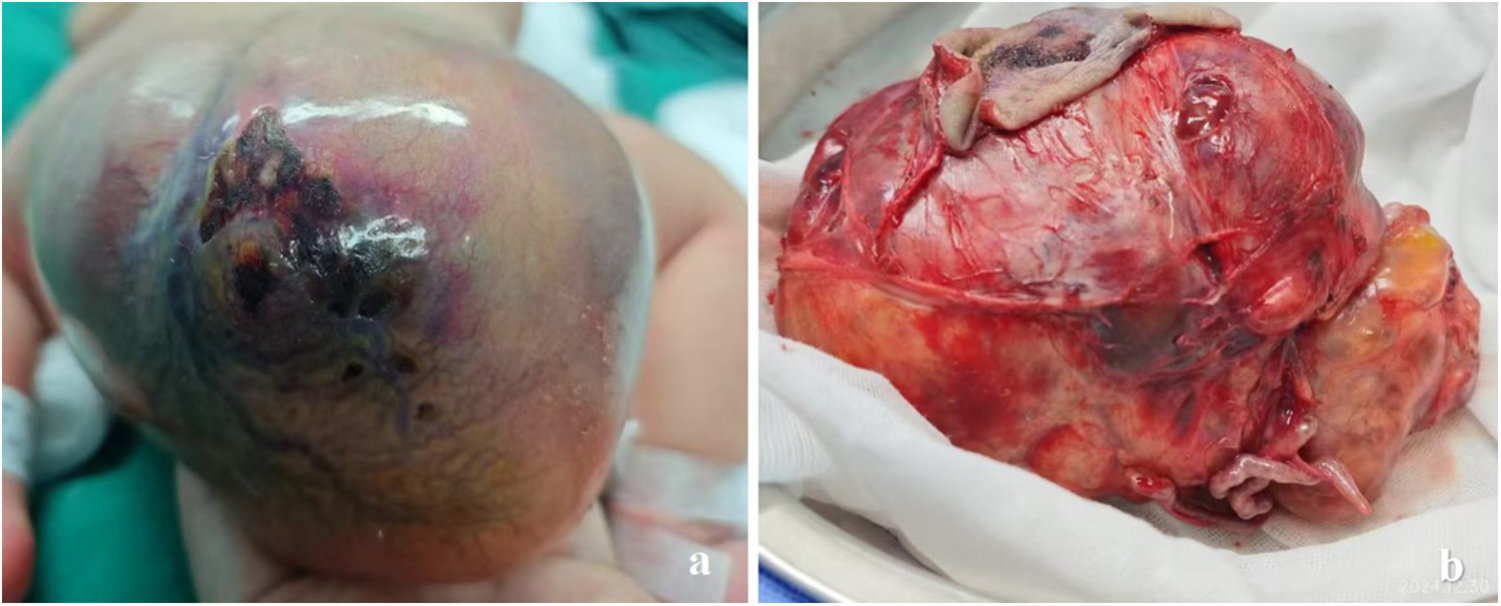
In case 3, **(a)** a neonate presented with a giant sacrococcygeal teratoma, with the mass resembling a head in appearance. **(b)** The appearance of the sacrococcygeal teratoma after complete resection, with two prominent vessels connected to the tumor tissue.

The infant was born at 38 weeks and 4 days, with a birth weight of 2,900 g, and a mass of approximately 12 cm × 12 cm × 10 cm was observed in the sacrococcygeal region (Figure 4a). Intraoperatively, two prominent vessels connecting to the tumor, measuring approximately 0.3 cm in diameter, were identified. These vessels were ligated, and the tumor was completely excised (Figure 4b). No abnormalities were observed in the sacrococcygeal ultrasound performed at 4 months postoperatively.

## Discussion

SCT is the most common tumor in neonates. The reported perinatal mortality rate varies widely in the literature, ranging from 13% to 50% (3). In prenatal diagnosis, beyond evaluating the placenta, amniotic fluid volume, and intrauterine fetal development, particular emphasis should be placed on identifying perinatal complications associated with fetal SCT (6). Prenatal complications such as hydrops with high-output cardiac failure, tumor necrosis and hemorrhage, polyhydramnios, preterm labor, and, in some cases, fetal demise can occur (7, 8). The risk of mortality increases significantly under these conditions; thus, serial monitoring with an ultrasound and echocardiography during pregnancy is essential (8, 9). On an ultrasound, signs such as placentomegaly, reversed diastolic blood flow in the umbilical arteries, and fluid accumulation in body cavities may indicate the onset of fetal hydrops (8, 9). These complications require prompt intervention, with a careful balance between urgent and planned management (8). It is crucial to assess the tumor size, composition, and vascularity to facilitate the formulation of an early surgical intervention plan (6). To mitigate the risk of hemorrhage, cesarean section should be considered for infants with large or highly vascularized tumors (10, 11).

The prognosis of a fetus or newborn diagnosed with a sacrococcygeal teratoma is determined by three key factors: the surgical resectability of the teratoma, the timing of the diagnosis, and the tumor's tissue composition (12, 13). Large and rapidly growing sacrococcygeal teratomas with substantial vascularity are linked to higher perinatal mortality and morbidity when compared to smaller, less vascularized lesions (14). The solid component of a sacrococcygeal teratoma typically demonstrates significant vascularity and a propensity for rapid expansion, thereby increasing the risk of massive hemorrhage and high-output cardiac failure (15). Prenatal surgical procedures for SCT lack evidence from randomized clinical trials, so patients should be selected carefully, with a postnatal intervention favored (16). Large sacrococcygeal tumors should be removed as soon as possible after birth to prevent rupture or intratumoral bleeding (16). In our cases, we performed surgery on the children within 48 h.

When evaluating a sacrococcygeal teratoma, the patient’s preoperative serum AFP level is a valuable diagnostic tool (17, 18). However, caution must be exercised when interpreting AFP levels, particularly in neonates and infants, as their natural hepatic production results in elevated concentrations. Therefore, relying solely on AFP levels cannot unequivocally confirm or exclude the presence of sacrococcygeal teratoma. Following the surgical resection of a sacrococcygeal teratoma, monitoring serum AFP levels can assist in detecting potential recurrences. It is crucial to interpret AFP levels in conjunction with other clinical findings and imaging studies for an accurate assessment (19). In our case series, one case demonstrated a gradual normalization of AFP levels following surgery but was found to have a recurrence during follow-up. Follow-up ultrasonography in the other two children showed no evidence of recurrence after surgery. Their AFP levels gradually decreased but remained elevated, which may be attributed to the relatively short duration of postoperative follow-up (currently only 4 months).

Coccygectomy is considered the optimal approach to prevent benign teratoma recurrence while minimizing damage to the tumor cyst wall. However, sacrococcygeal teratomas can recur years after complete excision, necessitating lifelong monitoring. Although survival rates are generally excellent, mortality for tumors larger than 10 cm can reach 18%. Even with complete coccygectomy, recurrence rates range from 11% to 22%, mandating close follow-up—consisting of a physical examination (including a rectal examination), diagnostic imaging, and AFP measurements—every 3–6 months for at least 3 years (20). In our case series, one patient experienced recurrence 1 year after the initial surgery and was successfully treated with a subsequent operation, showing no recurrence at the 2-year follow-up. The second patient remained recurrence-free at the 4-month follow-up.

Given the tumor's distinctive location, both bladder and rectal functions are widely recognized as being vulnerable to impairment from the tumor itself, surgical intervention, or both. Existing data indicate that bladder dysfunction may be underreported in individuals with benign sacrococcygeal teratoma (21). In our case series, one patient was found to have persistent congenital megacolon after surgery, without any observed bladder dysfunction. The other two patients showed no evidence of rectal or bladder dysfunction following surgery.

Neonatal hypothermia is also a concern during surgical procedures due to neonates’ low subcutaneous fat and immature thermoregulation, which makes them especially susceptible to intraoperative hypothermia. Before the neonate enters the operating room, it is essential to appropriately raise the room temperature and place an inflatable warming blanket on the surgical bed. Intraoperatively, the neonate's vital signs should be closely monitored. In all three of our cases, no intraoperative hypothermia was observed.

Prenatal ultrasound plays a crucial role in the management of sacrococcygeal teratomas, not only in the early detection and evaluation of the tumor's nature but also in the continuous monitoring of tumor growth and prenatal multidisciplinary team (MDT) interventions (22). Regular prenatal ultrasound examinations allow for precise measurement of the tumor's location and size (22). Based on the findings from a prenatal ultrasound and fetal MRI monitoring, an MDT consisting of pediatric surgeons, fetal medicine specialists, neonatologists, ultrasonographers, and obstetricians can assess each case individually, decide on the mode and timing of delivery, and formulate a postnatal treatment plan. This approach shortens the time required for a postnatal diagnosis and treatment in affected infants (22). The involvement of an experienced multidisciplinary team is crucial for providing comprehensive care, as their collective expertise enables them to evaluate the risks and benefits of various management options and develop a tailored treatment plan (19).

## Conclusion

Giant neonatal sacrococcygeal teratomas are rare, high-risk tumors associated with significant operative morbidity and mortality. We presented three neonatal cases, outlining our diagnostic and management strategies. Given the disease's rapid progression, clinicians must prepare comprehensive contingency plans; interdisciplinary collaboration is vital to improve survival outcomes and reduce complications.

## Data Availability

The original contributions presented in the study are included in the article/Supplementary Material, further inquiries can be directed to the corresponding author.
